# Cardiopulmonary resuscitation practices in the Netherlands: results from a nationwide survey

**DOI:** 10.1186/s12913-019-4166-2

**Published:** 2019-05-24

**Authors:** Marc Schluep, Geertje Johanna Catharina van Limpt, Robert Jan Stolker, Sanne Elisabeth Hoeks, Henrik Endeman

**Affiliations:** 1 Department of Anaesthesia, Erasmus University Medical Centre, P.O. Box 2040, 3000CA Rotterdam, the Netherlands; 2 Department of Intensive Care Medicine, Erasmus University Medical Centre, Rotterdam, the Netherlands

**Keywords:** Resuscitation care, Cardiopulmonary resuscitation, In-hospital cardiac arrest, Advanced life support, CPR practices

## Abstract

**Background:**

*S*urvival rates after in-hospital cardiac arrest are low and vary across hospitals. The ERC guidelines state that more research is needed to explore factors that could influence survival. Research into the role of cardiopulmonary resuscitation (CPR) practices is scarce. The goal of this survey is to gain information about CPR practices among hospitals in the Netherlands.

**Methods:**

A survey was distributed to all Dutch hospital organizations (*n* = 77). Items investigated were general hospital characteristics, pre-, peri- and post-resuscitation care. Characteristics were stratified by hospital teaching status.

**Results:**

Out of 77 hospital organizations, 71 (92%) responded to the survey, representing 99 locations. Hospitals were divided into three categories: university hospitals (8%), teaching hospitals (64%) and non-teaching hospitals (28%). Of all locations, 96% used the most recent guidelines for Advanced Life Support and 91% reported the availability of a Rapid Response System. Training frequencies varied from twice a year in 41% and once a year in 53% of hospital locations. The role of CPR team leader and airway manager is most often fulfilled by (resident) anaesthetists in university hospitals (63%), by emergency department professionals in teaching hospitals (43%) and by intensive care professionals in non-teaching hospitals (72%). The role of airway manager is most often attributed to (resident) anaesthetists in university hospitals (100%), and to intensive care professionals in teaching (82%) and non-teaching hospitals (79%).

**Conclusion:**

The majority of Dutch hospitals follow the ERC guidelines but there are differences in the presence of an ALS certified physician, intensity of training and participation of medical specialties in the fulfilment of roles within the CPR-team.

**Electronic supplementary material:**

The online version of this article (10.1186/s12913-019-4166-2) contains supplementary material, which is available to authorized users.

## Background

In-hospital cardiac arrest (IHCA) is a major adverse event for hospitalized patients with a reported incidence of 1.6/1000 admissions in European countries [1]. Cardiopulmonary resuscitation (CPR) is started to restore circulation, but survival rates are low and vary across various countries and hospitals [2–4]. Recent guidelines call for research on long-term and patient-centred outcomes, as well as strategies to help improve survival of IHCA [5, 6]. Much research is done on early recognition of patients at risk for IHCA and post-resuscitation care. The role of resuscitation practices itself and inter-hospital differences is far less examined, but is a growing focus in current research [7–9].

Survival after out-of-hospital cardiac arrest is relatively high in the Netherlands and we have learned from large cohort studies that optimisation of pre-hospital resuscitation care leads to higher survival [10]. The current European Resuscitation Council guidelines have several recommendations with regard to life support training of healthcare workers and strategies for prevention of cardiac arrest through Rapid Response Teams (RRT) [6]. Previous studies have demonstrated that quality characteristics such as team training and adherence to Advanced Life Support (ALS) guidelines are related to a higher survival probability for patients [9]. Furthermore hospital characteristics like teaching status, size and urban location are associated with differences in mortality after IHCA [11–15]. Knowledge about CPR practices might be useful for further research to optimise the chain of survival for IHCA.

The Resuscitation Outcome in the Netherlands (ROUTiNE) project is a nationwide initiative aimed at describing outcome of IHCA. Part of this project is a survey on CPR characteristics focussed on use of guidelines, training and organisation in Dutch hospitals. The goal of this survey is to describe the current resuscitation practices in the Netherlands.

## Methods

### Study population

A total of 77 Dutch hospital organizations with inpatient care facilities were identified in October 2017 by checking the report of the Dutch Hospitals Association (NVZ) on recent hospital mergers and acquisitions. In the Netherlands some hospitals are part of a larger organisation, but consist of different locations with independent facilities.

### Measures and data collection

A letter was sent to each board of directors to inform them about this study and to announce our survey. We acquired contact information of the local resuscitation coordinator or CPR committee chairperson via the database of the Dutch Association of Resuscitation Team Coordinators (NVCR) or by calling the hospital directly. A web-based survey was distributed to all Dutch hospital organizations from October 2017 to February 2018. The survey was completed by the resuscitation coordinator, a designated medical specialist or a CPR committee chairperson. Respondents were asked to fill out the survey for each hospital location with inpatient care facilities within their organization. Queries were performed for discrepancies and missing values and completed through reminder mailings.

The survey was developed based on literature [11] and contributions by CPR experts from in and outside the Erasmus University Medical Centre. Several multidisciplinary meetings were held in which we consulted with anaesthetists, cardiologists, intensive care specialists, a nurse resuscitation officer and an epidemiologist. No specific predesigned survey instrument was available, therefore we created our own short-form questionnaire focussed on CPR practices as reported by the designated specialists. Before its acquisition period, a pilot was held by the participants of the ROUTINE study (*n* = 18) to test clarity and comprehensiveness. Only minor adjustments were made to improve legibility.

This nationwide survey samples resuscitation practices with the goal of providing insights of resuscitation protocols at the institute. As no patient related data have been collected a formal IRB approval or waiver was not required to perform this survey. The online survey was built in LimeSurvey and included 63 items on 4 categories: general hospital characteristics, pre-, peri- and post-resuscitation care. We used dichotomous, multiple choice or multiple response questions for each item. Teaching hospitals were defined as providing medical specialty (registrar) training for at least two clinical specialties with an inpatient care facility, acknowledged by the Medical Specialist Registration Committee (RGS). Hospital geographic locations (i.e. metropolitan area, urban area, rural area) were determined by using the database of the Netherlands Environmental Assessment Agency (PBL).

Pre-resuscitation care involved preventive measures such as a Rapid Response Systems (RRS) and mandatory DNR-counselling upon admission. An RRS consists of an afferent component, also known as the track-and-trigger system, and an efferent component, the Rapid Response Team (RRT). Peri-resuscitation care was regarded as care provided when cardiac arrest had occurred and pertains to team constitutions, training level and frequency and guideline adherence. A multiple response question with more than one answer per participant was provided to indicate all possible CPR team members and their roles, as we assumed that roles are interchangeable and often depend on local agreements, the moment and location of the resuscitation. RRT or CPR team members were presented by medical specialty and by professional level. Professional level is divided in three groups: medical specialists, residents and nurses/paramedics, wherein residents also include house officers. Questions about post-resuscitation care pertained mainly to treatment strategies, intensive care availability and temperature management. Intensive care units are divided into three levels according to the National Dutch Intensive Care Guidelines. A summary of the specifications of intensive care levels can be found in Additional file 1: Appendix 1.

### Statistical analysis

All data were descriptive and presented as absolute numbers and percentages. For each variable with missing answers the number of respondents is mentioned at the sub header and the percentage is given relative to the available answers. Data are presented stratified by hospital teaching status; university hospitals, teaching hospitals and non-teaching hospitals. Analysis was done for hospital locations, unless otherwise stated.

### Ethical considerations

This research does not include any patients. Questions were distributed among colleagues from other hospitals in the Netherlands. All respondents consented to participation and publication of the results. No specific legislation applies.

## Results

Of the 77 hospital organisations, 71 (92%) hospital organisations responded to the survey. These hospitals represent 99 hospital locations.

### General hospital characteristics

Of the 99 hospital locations, 8 (8%) were university hospital locations, 63 (64%) were teaching hospitals and 28 (28%) were non-teaching hospitals. Table 1 shows the hospital characteristics. All university hospital locations had a level 3 Intensive Care Units (ICU), ICU’s of teaching hospital locations were mostly level 2 units (59%) and ICUs of non-teaching hospital locations were mostly classified as level 1 (75%). Of all hospital locations, 80 (81%) locations reported having a Coronary Care Unit (CCU). At organizational level, 29 (41%) hospital organizations reported having the ability to perform interventional cardiac catheterization and 16 (23%) reported having the ability to perform thoracic surgery.

**Table 1:** Hospital characteristics

Hospital location level n^a^ (%)	University locations (*n* = 8)	Teaching locations (*n* = 63)	Non-teaching locations (*n* = 28)	Total locations (*n* = 99)
Hospital size				
< 300 beds	0	25 *(39.7)*	21 *(75.0)*	46 *(46.5)*
300–600 beds	1 *(12.5)*	30 *(47.6)*	7 *(25.0)*	38 *(38.4)*
> 600 beds	7 *(87.5)*	8 *(12.7)*	0	15 (*15.2)*
Location				
Metropolitan area	4 *(50.0)*	24 *(38.1)*	9 *(32.1)*	37 *(37.4)*
Urban area	4 *(50.0)*	28 *(44.4)*	3 *(10.7)*	35 (*35.4)*
Rural area	0	11 *(17.5)*	16 *(57.1)*	27 *(27.3)*
Availability				
Mobile Cardiac Telemetry	8 *(100)*	50 *(79.4)*	24 *(85.7)*	82 *(82.8)*
Coronary Care Unit	8 *(100)*	48 *(76.2)*	24 *(85.7)*	80 *(80.8)*
Medium Care or High Care	8 *(100)*	29 *(46.0)*	6 *(21.4)*	43 *(43.3)*
Intensive Care Unit	8 *(100)*	49 *(77.8)*	24 *(85.7)*	81 *(81.8)*
Level 1	0	7/49 *(14.3)*	18/24 *(75.0)*	24/81 *(29.6)*
Level 2	0	29/49 *(59.2)*	5/24 *(20.8)*	34/81 *(42.0)*
Level 3	8/8 *(100)*	13/49 *(26.5)*	1/24 *(4.2)*	22/81 *(27.2)*
Emergency Room	8 *(100)*	53 *(84.1)*	25 *(89.3)*	86 *(86.8)*
24/7	8/8 *(100)*	49/53 *(92.5)*	24/25 *(96.0)*	81/86 *(94.2)*
Daytime + evening	0	2/53 *(3.8)*	1/25 *(4.0)*	3/86 *(3.5)*
Daytime	0	2/53 *(3.8)*	0	2/86 *(2.3)*
Hospital organisational level	University organizations (*n* = 8)	Teaching organizations (*n* = 39)	Non-teaching organizations (*n* = 24)	Total organizations (*n* = 71)
Availability				
Trauma Centre	8 *(100)*	3 *(7.7)*	0	11 *(15.5)*
Abdominal aortic surgery	8 *(100)*	31 *(79.5)*	12 *(50.0)*	51 *(71.8)*
Neurosurgery	8 *(100)*	9 *(23.1)*	0	17 *(23.9)*
Thoracic surgery	8 *(100)*	8 (*20.5)*	0	16 *(22.5)*
Interventional Cardiac Cath.	8 *(100)*	20 *(51.3)*	1 *(4.2)*	29 *(40.8)*

^a^In case of missing values or other denominator than all hospitals, the denominator is given

### Pre-resuscitation care

Table 2 provides an overview of the characteristics of the pre- and peri-resuscitation care across the different hospital types. DNR-counselling upon admission was reported as mandatory in 88 (89%) of all hospital locations. Most locations reported having an RRS (91%), of which one hospital reported an RRS without the use of the efferent component, an RRT. The Modified Early Warning System (MEWS) was the most frequently used track-and-trace system in university hospital locations (71%), while the Early Warning Score (EWS) was reported to be most used in teaching and non-teaching hospitals. It has to be stated that the term EWS can be used in the Netherlands for both using the old (binary) EWS, but also for the MEWS. In 55 (65%) hospital locations the RRT consisted of two persons. More details about pre-resuscitation care can be found in Additional file 1: Table S4.

**Table 2:** Pre- and peri-resuscitation care characteristics

Hospital location level n^a^ (%)	University locations (*n* = 8)	Teaching locations (*n* = 63)	Non-teaching locations (*n* = 28)	Total locations *n* = 99)
Pre-arrest variables				
Mandatory DNR-counselling upon admission	8 *(100)*	57 *(90.5)*	23 *(82.1)*	88 *(88.9)*
Rapid Response System available	7 *(87.5)*	55/61 *(90.2)*	25/27 *(92.6)*	87/96 *(90.6)*
Type of Rapid Response System				
EWS	2/7 *(28.6)*	33/55 *(60.0)*	16/25 *(64.0)*	51/87 *(58.6)*
MEWS	5/7 *(71.4)*	13/55 *(23.6)*	8/25 *(32.0)*	26/87 *(29.9)*
NEWS	0	2/55 *(3.6)*	1/25 *(4.0)*	3/87 *(3.4)*
Own scoring system	0	5/55 *(9.1)*	0	5/87 *(5.7)*
Number of team members RRT				
2 persons	6/6 *(100)*	34/54 *(62.9)*	15/25 *(60.0)*	55/85 *(64.7)*
3 persons	0	15/54 *(27.8)*	9/25 *(36.0)*	23/85 *(27.1)*
4 persons	0	2/54 *(3.7)*	1/25 *(4.0)*	3/85 *(3.5)*
Peri-arrest variables				
ERC/NRR 2015 Guidelines	8 *(100)*	60 *(95.2)*	27 *(96.4)*	95 *(96.0)*
Availability Medical Doctor with ALS certificate				
24/7	8 *(100)*	37/62 *(59.7)*	11 *(39.3)*	56/98 *(57.1)*
Daytime + evening	0	4/62 *(6.5)*	3 *(10.7)*	7/98 *(7.1)*
Daytime	0	3/62 *(4.8)*	0	3/98 *(3.1)*
No strict regulations	0	12/62 *(19.4)*	10 *(35.7)*	22/98 *(22.4)*
No doctor with ALS certificate	0	6/62 *(9.7)*	4 *(14.3)*	10/98 *(10.2)*
Training frequency				
Twice a year	2 *(25.0)*	26 *(41.3)*	13 *(46.4)*	41 *(41.4)*
Once a year	4 *(50.0)*	35 *(55.5)*	13 *(46.4)*	52 *(52.5)*
Less than once a year	2 *(25.0)*	2 *(3.2)*	2 *(7.1)*	6 *(6.1)*
Transthoracic echo use during CPR	7 *(87.5)*	35/62 *(56.5)*	17 *(60.7)*	59/98 *(60.2)*
Performed by (resident) cardiologist	7/7 *(100)*	26/35 *(74.3)*	14/17 *(82.4)*	47/59 *(79.7)*
Mechanical CPR use during CPR	5 *(62.5)*	23 *(36.5)*	12 *(42.9)*	39 *(39.4)*
LUCAS	2/5 *(40.0)*	15/23 *(65.2)*	9/12 *(75.0)*	27/39 *(66.7)*
AutoPulse	2/5 *(40.0)*	6/23 *(26.1)*	3/12 *(25.0)*	11/39 *(28.2)*
Team size				
< 4 persons	2 *(25.0)*	10/59 *(16.9)*	5/26 *(19.2)*	17/93 *(18.3)*
4 persons	3 *(37.5)*	17/59 *(28.8)*	3/26 *(11.5)*	23/93 (*24.7)*
> 4 persons	3 *(37.5)*	32/59 *(54.2)*	18/26 (*69.2)*	53/93 (*57.0)*

^a^In case of missing values or other denominator than all hospitals, the denominator is given. *DNR* Do Not Resuscitate, *(M/N)EWS* (Modified/National) Early Warning System, *RRT* Rapid Response Team, *ERC* European Resuscitation Council, *NRR* Dutch resuscitation council, *ALS* Advanced Life Support, *CPR* Cardiopulmonary Resuscitation, *LUCAS* Lund University Cardiopulmonary Assist System

### Peri-resuscitation care

**Fig. 1:**
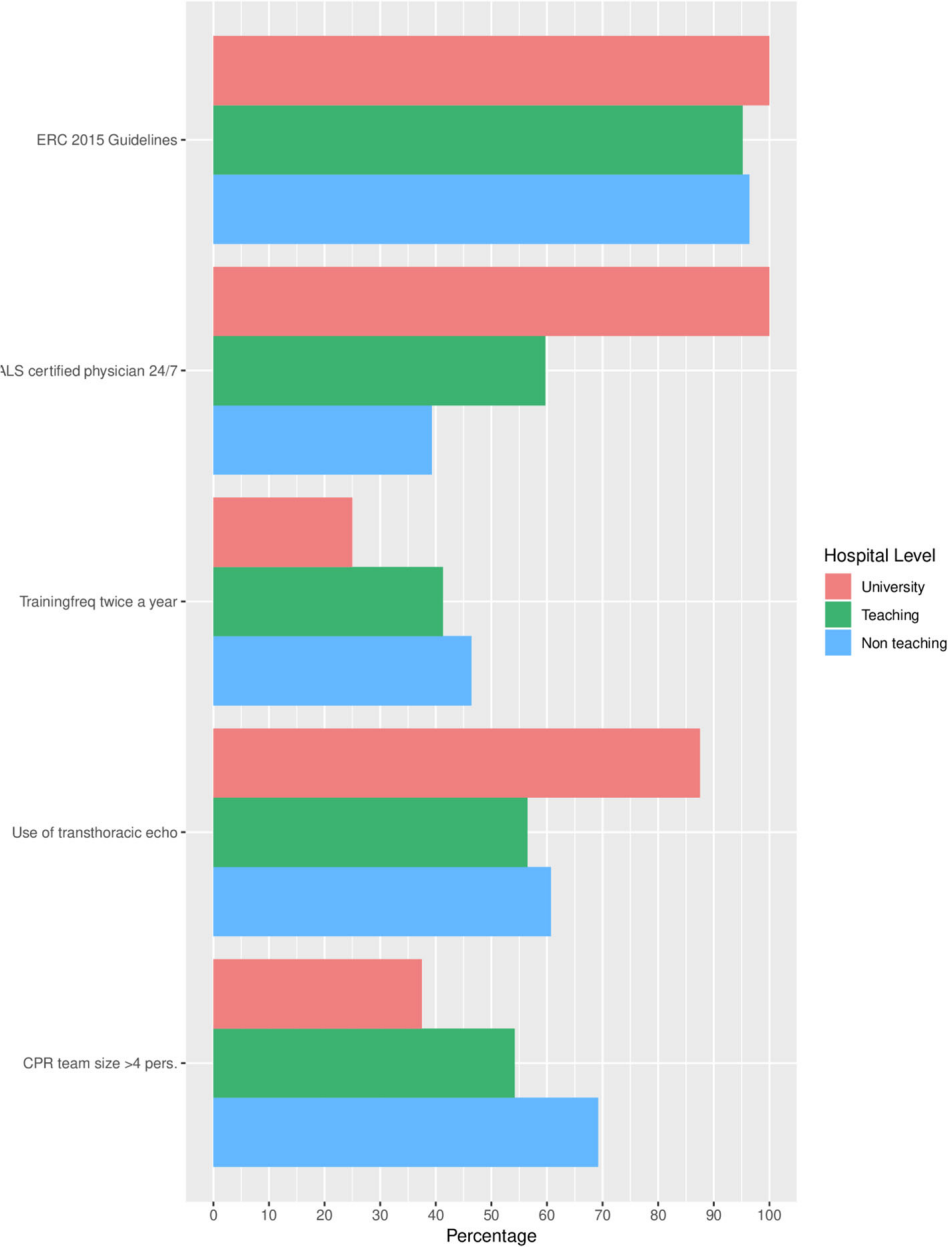
Resuscitation characteristics by hospital level. ERC, European resuscitation counsel; ALS, advanced life support; CPR, cardiopulmonary resuscitation

The second portion of Table 2 summarizes the equipment, structure and organization of peri-resuscitation care across all responding hospital locations and Fig. 1 shows the distribution of these characteristics by hospital type. Ninety-five (96%) hospital locations reported following the 2015 ERC guidelines. All university hospital locations reported an ALS certified medical doctor available in the team 24/7. In teaching and non-teaching hospital locations this was the case in 37 (60%) and 11 (40%) locations respectively. A total of 22 (22%) hospital locations reported the availability of an ALS certified medical doctor as not strictly regulated in their hospital and 10 (10%) locations reported not having ALS certified medical doctors employed.

Training frequencies of the CPR team members varied across the hospital locations with 41 (41%) training twice a year, 52 (53%) training once a year and 6 (6%) hospitals training less than once a year. Teaching and non-teaching hospital locations reported to offer CPR training with a minimal of twice a year (41 and 46% respectively) more often in comparison with university hospitals (25%). Transthoracic echo use during CPR is reported by 59 (60%) of all hospital locations, performed mainly by a cardiologist or resident cardiology (80%). CPR teams consisted of minimal five team members in three (38%) university hospital locations, 32 (54%) teaching hospital locations and 18 (69%) non-teaching hospital locations.

**Fig. 2:**
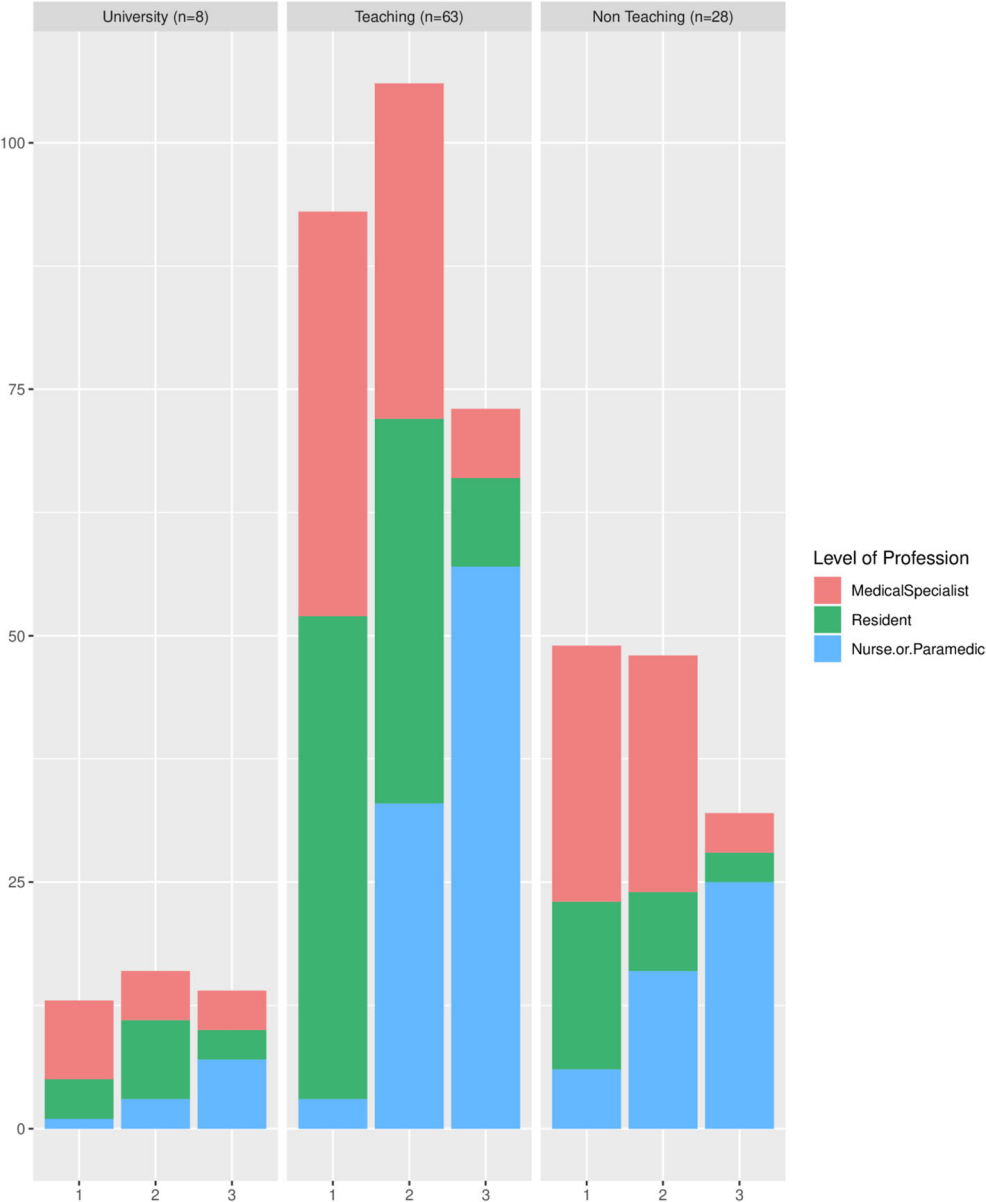
Constitution CPR teams by level of profession. Role in CPR team (1 = Team leader, 2 = Airway man., 3 = Circulation manager)

**Fig. 3:**
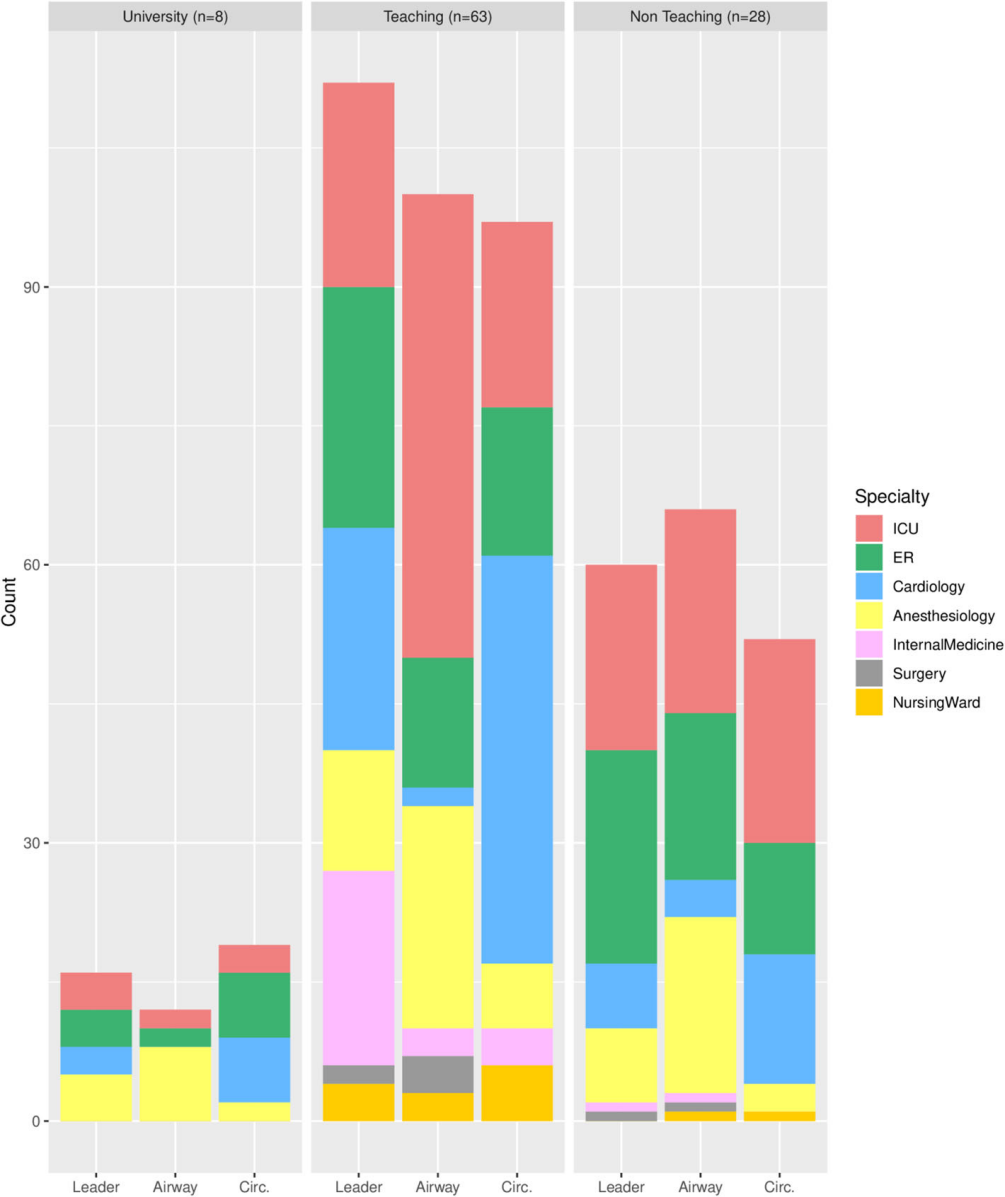
Constitution CPR teams by medical specialty. CPR roles are displayed below

The absolute frequency distribution of team leader, airway manager and circulation manager roles are shown in Fig. 2 (by level of profession) and Fig. 3 (by medical specialty) and specified in Additional file 1: Table S5. In teaching hospital locations, residents were reported more often as fulfilling the role of team leader during CPR than medical specialists (80% versus 67%). In university hospital locations (resident) anaesthetists were most often mentioned (in 63% of the cases), when in teaching hospital locations this role assignment was more often attributed to intensive care (36%), emergency care (43%) cardiology (39%) and internal medicine (34%). In non-teaching hospital locations, physicians from the intensive care and emergency department were identified most often (71 and 82% respectively) as being team leader during CPR. This pattern was also seen for the role of airway manager. All respondents of the university hospital locations identified the (resident) anaesthetist as airway manager, while teaching and non-teaching hospital locations identified the intensive care physician mostly (82 and 79% respectively) as responsible for this role.

## Discussion

This nationwide survey covering 92% of all Dutch hospital organizations shows that CPR practices differ between hospitals. Although almost all hospital locations reported following the most current European guidelines for ALS, there are differences between hospitals in CPR training frequencies, the availability of ALS certified medical doctors during day and night and constitution of the CPR teams.

The current European Resuscitation Council Guidelines recommend a CPR (re) training more than once a year, because ALS knowledge and skills deteriorate within 6 to 12 months after ALS training [6, 16]. Previous studies showed improved survival from IHCA when the responding emergency team included ALS-trained individuals [17–19]. In our study, 94% of all hospital locations report that CPR team members received routine resuscitation training, but only 57% of all hospitals reported round the clock availability of an ALS certified medical doctor. Ten per cent reported not having ALS certified medical doctors employed, although we do not know if other training was provided.

The importance of CPR practices have not yet been fully elicited, however a recent publication finds that top-performing hospitals with regard to survival rates after IHCA show several common practices [9]. The first is a formally organized team composed of members from diverse disciplines. These members had delineated roles and responsibilities. They speak of strong communication, leadership and a focus on training and education. In our own study these features are clearly depicted. All responding hospitals have a designated CPR team. In general there is no formal organization of CPR teams, however they always consist of medical professionals (residents or specialists) who are trained a field of acute care. As this was not a qualitative study, we cannot make statements about strong communication. We can state that training and education is mostly according to ERC guidelines and 94% of hospitals train their personnel at least once a year.

In previous self-reported survey studies, conducted in 2009 and 2015 in the US, Germany, Austria and Switzerland, only 52–62% of hospitals reported offering routine training for CPR team members [20, 21]. Another survey in the UK showed that 49% of junior doctors participating in CPR team not had ALS training, but would like to do so. These differences with our findings may be due to the fact that surveys of Siebig et al. and Morgan et al. were anonymous [21, 22]. Furthermore, the routine use of resuscitation officers in the Netherlands may contribute to this difference. This is in line with the survey of Edelson, who stated that less than half of the responding hospitals reported the presence of a resuscitation officer, which is correlated with routine CPR training [20]. Our study shows that CPR teams trained more than once a year in 41% of the cases, which is in line with our neighbouring countries [21].

Our results showed that CPR team physicians consisted of cardiologists, anaesthetists, intensivists and physicians from the emergency department. A German survey from 2009 reported that 80% of the physicians in the resuscitation team worked on an intensive care. Furthermore they reported that 55% of the physicians in the CPR team had a specialist qualification [21]. A survey conducted in the UK, reported the team leader is in 73–82% of cases represented by an emergency consultant [23]. In the present study we found comparable results: in the roles of team leader, airway manager and circulation manager the participation of residents and medical specialist was almost equally distributed. When stratified by hospital type, residents were most often participating in CPR teams in university hospitals and least often in non-teaching hospitals. This corresponds with findings of Edelson et al. [20]. An exploratory study showed that junior physicians are competent overall in managing resuscitation attempts but partly failed in the role of team leader [24]. We found a relatively low number of anaesthetists performing airway management. This finding should be elicited by the fact that intensive care medicine in the Netherlands is performed by anaesthetists or internists with a focus in ICU-medicine, Based on local protocol airway management service is therefore provided by either the department of anaesthesia or the ICU. The total number of team members per CPR team in our survey is comparable with results reported by Porter el al., who stated resuscitation teams consisted in 64–69% of cases of four to six members [23].

In our study, 91% of the responding hospital locations reported to have a Rapid Response System (RRS) in place, which is in line with previous Dutch studies and also with findings of a survey in the US [20, 25]. However, in 2008, the implementation of RRS was mandated by the Dutch government. Reasons for not having an RRS implemented yet are unclear.

### Strengths and limitations

A limited amount of studies has investigated and described in-hospital CPR care. A key strength of our study is that, to our knowledge, this is the first Dutch survey of in-hospital practices that covers pre-, peri- and post-resuscitation care. We obtained a high response rate. However, it has to be taken in account that we gathered data through a non-anonymous self-report method, which could have negatively influenced the reliability of our data. Thereby, the questions mainly pertain to mandatory characteristics, which could have led to a risk of reporting bias and answers based on organisational policy instead of actual practice. We assessed availability of an ALS-certified physician during day- and night-time rather than the round the clock variance in team constitution. We presumed this to be a proxy of team training level. Lastly, one of the previously identified predictors of better outcome could not be obtained from this survey, i.e. debriefings after CPR attempts [11, 26]. The reason is that this is mostly not documented.

This was a descriptive study and we did not investigate survival rates or other patient related outcomes. We conclude there is some variability across hospitals in the Netherlands. Protocol adherence and training frequencies are adequate. We aim to combine these data with our survival figures from the Resuscitation Outcomes in the Netherlands – project to better assess factors that influence survival.

## Conclusion

The majority of Dutch hospitals follow the ERC guidelines but there are differences in the presence of an ALS certified physician, intensity of training and participation of medical specialties in the fulfilment of roles within the CPR-team. Knowledge on resuscitation practices and learning from best practice can be useful in improving CPR quality and can be of interest in future research.

## Additional file

Additional file 1: **Appendix 1.** Specifications of Intensive Care levels according to the National Dutch Intensive Care guideline 2006 [21,37]. **Table S4** Pre- and post-resuscitation care characteristics (cont’d). *In case of missing values or other denominator than all hospitals, the denominator is given. **Table S5** Roles of resuscitation team participants. *In case of missing values or other denominator than all hospitals, the denominator is given. (DOCX 33 kb)

## Data Availability

The dataset from this study is freely available from the corresponding author upon request.

## Abbreviations

ALS: Advanced Life Support
CCU: Coronary/cardiac care unit
CPR: Cardiopulmonary Resuscitation
DNR: Do Not Resuscitate
ERC: European Resuscitation Council
EWS: Early Warning System
ICU: Intensive Care Unit
IHCA: In-Hospital Cardiac Arrest
NVCR: Nederlandse Vereniging voor (intramurale) Coördinatoren Reanimatie
NVZ: Nederlandse Vereniging van Ziekenhuizen
PBL: PlanBureau voor de Leefomgeving
ROUTINE: Resuscitation Outcomes in the Netherlands
RRS: Rapid Response System
RRT: Rapid Response Team
