# Supplementary material for: Cardiopulmonary resuscitation practices in the Netherlands: results from a nationwide survey
Source: BMC Health Serv Res. 2019 May 24;19:333. doi: 10.1186/s12913-019-4166-2 (PMC6534892; doi:10.1186/s12913-019-4166-2)
Supplement: Supplementary file 1 — Appendix 1. Specifications of Intensive Care levels according to the National Dutch Intensive Care guideline 2006 [21,37]. Table S4 Pre- and post-resuscitation care characteristics (cont’d). *In case of missing values or other denominator than all hospitals, the denominator is given. Table S5 Roles of resuscitation team participants. *In case of missing values or other denominator than all hospitals, the denominator is given. (DOCX 33 kb) [file 12913_2019_4166_MOESM1_ESM.docx]

**APPENDIX 1**

Specifications of Intensive Care levels according to the National Dutch Intensive Care guideline 2006[21,37].

| **Criteria** | **Intensive Care**  **level 3** | **Intensive Care**  **level 2** | **Intensive Care**  **level 1** |
| --- | --- | --- | --- |
| **Head** | Intensivist | Intensivist | Intensivist |
| **Medical responsibility** | Intensivist | Intensivist |  |
| **Procedural responsibility** | Intensivist coordinates care and makes formal arrangements on local level so that other specialists can take their own responsibility. | Intensivist coordinates care and makes formal arrangements on local level so that other specialists can take their own responsibility. | Intensivist coordinates care and makes formal arrangements on local level so that other specialists can take their own responsibility. |
| **Continuity of care, daytime** | 7d/week | 7d/week | 5d/week |
| **Continuity of care, night time** | Exclusively on call, within 20 min. at bedside. IC house officer within 5 min. at bedside | Exclusively on call, within 20 min. at bedside. IC house officer within 5 min. at bedside | On call, within 2h at bedside. IC house officer within 5 min. at bedside |
| **Intensivist staffing (Full Time Equivalents = FTE)** | 0,45 - 0,55 / bed | 0,35 - 0,42 / bed | 0,1 - 0,15 / bed |
| **ICU house officer staffing (FTE)** | 0,6 - 0,9 / bed | 0,55 / bed | 5-6 / hospital |
| **IC nurse staffing (FTE)** | 4,2 / ventilation bed | 3,5 / ventilation bed | 2,7 / ventilation bed |
| **Treatment days / year** | >3000 | > 2500 |  |
| **Ventilation days / year** | >1500 | > 1250 |  |
| **Optimal size ICU** | 12 beds | 12 beds | 6 beds |
| **Optimal size subunit** | 6-12 beds | 6-12 beds | 6-12 beds |
| **Quality** | Quality system, annual report | Quality system, annual report | Quality system, annual report |
| **Indicators** | Minimal set quality indicators for internal use | Minimal set quality indicators for internal use | Minimal set quality indicators for internal use |
| **Evaluation medical treatment** | Complication- and necrology conference | Complication- and necrology conference | Complication- and necrology conference |
| **Interdisciplinary counselling** | Daily multidisciplinary patient conference | Daily multidisciplinary patient conference | Daily multidisciplinary patient conference |
| **Guidelines & protocols** | + | + | + |
| **Material** | Following scientific societies and European guidelines | Following scientific societies and European guidelines | Following scientific societies and European guidelines |
| **Regionalisation** | Participation in regional network, supra regional coordinating function | Participation in regional network | Participation in regional network, if expected ventilation > 3d: consultation of higher level IC within 24h |
| **Bed utilisation** | Secondary to regional needs | Secondary to regional needs |  |

| **Hospital location level**  **n* (%)** | **University locations (n=8)** | **Teaching locations (n=63)** | **Non-teaching locations (n=28)** | **Total**  **locations (n=99)** |
| --- | --- | --- | --- | --- |
| **Pre-arrest variables** | | | | |
| **Rapid Response Call recipient during office hours** | | | | |
| **By medical specialty** |  |  |  |  |
| **ICU** | 7/7 *(100)* | 40/50 *(80.0)* | 17/23 *(73.9)* | 64/80 *(80.0)* |
| **ER** | 0 | 7/50 *(14.0)* | 4/23 *(17.4)* | 11/80 *(13.8)* |
| **Other** |  | 3/50 *(6.0)* | 2/23 (*8.7)* | 5/80 *(6.3)* |
| **By professional level** |  |  |  |  |
| **Medical specialist** | 7/7 *(100)* | 10/50 *(20.0)* | 9/23 *(39.1)* | 26/80 (*32.5)* |
| **Resident** | 0 | 17/50 *(34.0)* | 2/23 *(8.7)* | 19/80 (*23.8)* |
| **Nurse/Paramedic/other** | 0 | 23/50 *(46.0)* | 12/23 *(52.2)* | 35/80 (*43.8)* |
| **Rapid Response Call recipient outside office hours** | | | | |
| **By medical specialty** |  |  |  |  |
| **ICU** | 6/7 *(100)* | 41/52 *(78.8)* | 17/24 *(68.0)* | 64/83 *(77.1)* |
| **ER** | 0 | 8/52 *(15.4)* | 7/24 *(28.0)* | 15/83 *(18.1)* |
| **Other** | 0 | 3/52 *(5.8)* | 0 | 3/83 *(3.6)* |
| **By professional level** |  |  |  |  |
| **Medical specialist** | 6/7 *(100)* | 8/52 *(15.4)* | 7/24 *(29.2)* | 21/83 *(25.3)* |
| **Resident** | 0 | 21/52 *(40.4)* | 5/24 *(20.8)* | 26/83 *(31.3)* |
| **Nurse/Paramedic** | 0 | 21/52 *(40.4)* | 12/24 *(50.0)* | 33/83 *(39.8)* |
| **Target response time ward doctor** | | | | |
| **Within 15 minutes** | 0 | 12/51 *(19.6)* | 3/25 *(12.0)* | 15/83 *(18.1)* |
| **Within 15-30 minutes** | 5/7 *(71.4)* | 36/51 *(70.6)* | 18/25 *(72.0)* | 59/83 *(71.1)* |
| **Within >30 minutes** | 1/7 *(14.3)* | 1/51 *(2.0)* | 0 *(0)* | 2/83 *(2.4)* |
| **No strict regulations** | 1/7 *(14.3)* | 2/51 *(3.9)* | 4/25 *(16.0)* | 7/83 *(8.4)* |
| **Target response time RRT** | | | | |
| **Within 10 minutes** | 4/6 *(66.7)* | 31/52 *(59.6)* | 17/25 *(68.0)* | 52/83 *(62.7)* |
| **Within 15 minutes** | 2/6 *(33.3)* | 11/52 *(21.2)* | 6/25 *(24.0)* | 19/83 *(22.9)* |
| **Within 15-30 minutes** | 0 | 3/52 *(5.8)* | 1/25 *(4.0)* | 4/83 *(4.8)* |
| **No strict regulations / No RRT** | 0 | 7/52 *(13.5)* | 1/25 *(4.0)* | 8/83 *(9.6)* |
| **Profession of team members RRT** | | | | |
| **By medical speciality** |  |  |  |  |
| **Intensive Care** | 7/7 *(100)* | 48/52 *(92.3)* | 23/25 *(92.0)* | 78/84 *(92.9)* |
| **ER** | 0 | 12/52 *(23.1)* | 17/25 *(68.0)* | 29/84 *(34.5)* |
| **Anaesthesiology** | 0 | 2/52 *(3.8)* | 1/25 *(4.0)* | 3/84 *(3.6)* |
| **CCU** | 0 | 2/52 *(3.8)* | 2/25 *(8.0)* | 4/84 *(4.8)* |
| **By professional level** |  |  |  |  |
| **Medical specialist** | 7/7 *(100)* | 32/52 *(61.5)* | 22/25 *(88.0)* | 61/84 *(72.6)* |
| **Resident** | 2/7 *(28.6)* | 40/52 *(76.9)* | 17/25 *(68.0)* | 59/84 *(70.2)* |
| **Nurse/Paramedic** | 6/7 *(85.7)* | 51/52 *(98.1)* | 25/25 *(100)* | 82/84 *(97.6)* |
| **Post-arrest variables** |  |  |  |  |
| **Availability Intensive Care Unit** | 8 *(100)* | 49 *(77.8)* | 24 *(85.7)* | 81 *(81.8)* |
| **Intensivist physically present in ICU** |  |  |  |  |
| **24/7** | 7 *(87.5)* | 23/49 *(46.9)* | 5/24 *(20.8)* | 35/81 *(43.2)* |
| **Daytime + evening** | 1 *(12.5)* | 17/49 *(34.7)* | 7/24 *(29.2)* | 25/81 *(30.9)* |
| **Daytime** | 0 | 9/49 *(18.4)* | 11/24 *(45.8)* | 20/81 *(24.7)* |
| **Extracorporeal Life Support** | 7 *(87.5)* | 11/48 *(22.9)* | 0 | 18/79 *(22.8)* |
| **Availability Post Resuscitation Temperature Management** | 8 *(100)* | 48/48 *(100)* | 22/23 *(95.7)* | 79/80 *(98.8)* |
| **Post Resuscitation Target Temperature** | | | | |
| **33°C** | 3/7 *(42.9)* | 18/48 *(37.5)* | 7/22 *(31.8)* | 28/77 *(36.4)* |
| **36°C** | 3/7 *(42.9)* | 28/48 *(58.3)* | 13/22 *(59.1)* | 44/77 *(57.1)* |
| **33°C or 36°C** | 1/7 *(14.3)* | 2/48 *(4.2)* | 2/22 *(9.1)* | 5/77 *(6.5)* |
| **Cooling technique** | | | | |
| **Surface cooling (with target temperature)** | 5/8 *(62.5)* | 35/48 *(72.9)* | 18/22 *(81.8)* | 58/78 *(74.4)* |
| **Invasive (with target temperature)** | 2/8 *(25.0)* | 6/48 *(12.5)* | 1/22 *(4.5)* | 9/78 *(11.5)* |
| **Conventional (ice packs. cold infusion)** | 1/8 *(12.5)* | 7/48 *(14.6)* | 3/22 *(13.6)* | 11/78 *(14.1)* |

**Supplemental table 4** Pre- and post-resuscitation care characteristics (cont’d). *In case of missing values or other denominator than all hospitals, the denominator is given.

| **Hospital location level**  **n* (%)** | **University locations (n=8)** | **Teaching locations (n=63)** | **Non-teaching locations  (n=28)** | **Total**  **locations**  **(n=99)** |
| --- | --- | --- | --- | --- |
| **Team leader by medical specialty** | | | | |
| **Intensive Care** | 4 *(50.0)* | 22/61 *(36.1)* | 20 *(71.4)* | 46/97 *(47.4)* |
| **ER** | 4 *(50.0)* | 26/61 *(42.6)* | 23 *(82.1)* | 53/97 *(54.6)* |
| **Cardiology** | 3 *(37.5)* | 24/61 *(39.3)* | 7 *(25.0)* | 34/97 *(35.1)* |
| **Anaesthesiology** | 5 *(62.5)* | 13/61 *(21.3)* | 8 *(28.6)* | 26/97 *(26.8)* |
| **Internal Medicine** | 0 | 21/61 *(36.1)* | 1 *(3.6)* | 22/97 *(22.7)* |
| **Other** | 0 | 6/61 *(9.8)* | 1 *(3.6)* | 7/97 *(7.2)* |
| **Team leader by level of profession** | | | | |
| **Medical Specialist** | 8 *(100)* | 41/61 *(67.2)* | 26 *(92.9)* | 75/97 *(77.3)* |
| **Resident** | 4 *(50.0)* | 49/61 *(80.3)* | 17 *(60.7)* | 70/97 *(72.2)* |
| **Nurse/Paramedic** | 1 *(12.5)* | 3/61 *(4.9)* | 6 *(21.4)* | 10/97 *(10.3)* |
| **Airway manager by medical specialty** | | | | |
| **Intensive Care** | 2 *(25.0)* | 50/61 *(82.0)* | 22 *(78.6)* | 74/97 *(76.3)* |
| **ER** | 2 *(25.0)* | 14/61 *(23.0)* | 18 *(64.3)* | 34/97 *(35.1)* |
| **Cardiology** | 0 | 2/61 *(3.3)* | 4 *(14.3)* | 6/97 *(6.1)* |
| **Anaesthesiology** | 8 *(100)* | 24/61 *(39.3)* | 19 *(67.9)* | 51/97 *(52.6)* |
| **Internal Medicine** | 0 | 3/61 *(4.9)* | 1 *(3.6)* | 4/97 *(4.1)* |
| **Other** | 0 | 7/61 *(11.5)* | 2 *(7.1)* | 9/97 *(9.3)* |
| **Airway manager by level of profession** | | | | |
| **Medical Specialist** | 5 *(62.5)* | 34/61 *(55.7)* | 24 *(85.7)* | 63/97 *(64.9)* |
| **Resident** | 8 *(100)* | 39/61 *(63.9)* | 8 *(28.6)* | 55/97 *(56.7)* |
| **Nurse/Paramedic** | 3 *(37.5)* | 33/61 *(54.1)* | 16 *(57.1)* | 52/97 *(53.6)* |
| **Circulation manager by medical specialty** | | | | |
| **Intensive Care** | 3 *(37.5)* | 20/62 *(32.3)* | 22/27 *(81.5)* | 45/97 *(46.4)* |
| **ER** | 7 *(87.5)* | 16/62 *(25.8)* | 12/27 *(44.4)* | 35/97 *(36.1)* |
| **Cardiology** | 7 *(87.5)* | 44/62 *(71.0)* | 14/27 *(51.9)* | 65/97 *(67.0)* |
| **Anaesthesiology** | 2 *(25.0)* | 7/62 *(11.3)* | 3/27 *(11.1)* | 12/97 *(12.4)* |
| **Internal Medicine** | 0 | 4/62 *(6.5)* | 0 | 4/97 *(4.1)* |
| **Other** | 0 | 6/62 *(9.7)* | 1/27 *(3.7)* | 7/97 *(7.2)* |
| **Circulation manager by level of profession** | | | | |
| **Medical Specialist** | 4 *(50.0)* | 7/62 *(11.3)* | 4/27 *(14.8)* | 15/97 *(15.5)* |
| **Resident** | 3 *(37.5)* | 9/62 *(14.5)* | 3/27 *(11.1)* | 15/97 *(15.5)* |
| **Nurse/Paramedic** | 7 *(87.5)* | 57/62 *(91.9)* | 25/27 *(92.6)* | 89/97 *(91.8)* |
| **Chest compressor by level of profession** | | | | |
| **Medical Specialist** | 4 *(50.0)* | 6/60 *(10.0)* | 3 *(10.7)* | 13/96 *(16.7)* |
| **Resident** | 4 *(50.0)* | 11/60 *(18.3)* | 7 *(25.0)* | 22/96 *(22.9)* |
| **Nurse/Paramedic** | 7 *(87.5)* | 58/60 *(96.7)* | 27 *(96.4)* | 92/96 *(95.8)* |
| **IV/IO access + meds by level of profession** | | | | |
| **Medical Specialist** | 0 | 0 | 0 | 0 |
| **Resident** | 1 *(12.5)* | 7/59 *(11.9)* | 2/27 *(7.4)* | 10/94 *(10.6)* |
| **Nurse/Paramedic** | 7 *(87.5)* | 57/59 *(96.6)* | 27/27 *(100)* | 91/94 *(96.8)* |

**Supplemental table 5** Roles of resuscitation team participants. *In case of missing values or other denominator than all hospitals, the denominator is given.
